# Influence of FTO rs9939609 and Mediterranean diet on body composition and weight loss: a randomized clinical trial

**DOI:** 10.1186/s12967-018-1680-7

**Published:** 2018-11-12

**Authors:** Laura Di Renzo, Giorgia Cioccoloni, Simone Falco, Ludovico Abenavoli, Alessandra Moia, Paola Sinibaldi Salimei, Antonino De Lorenzo

**Affiliations:** 1 Section of Clinical Nutrition and Nutrigenomic, Department of Biomedicine and Prevention, University of Rome Tor Vergata, Via Montpellier 1, 00133 Rome, Italy; 2 PhD School of Applied Medical-Surgical Sciences, University of Rome Tor Vergata, Via Montpellier 1, 00133 Rome, Italy; 3 Department of Health Sciences, University of Magna Græcia, Viale Europa, Germaneto, 88100 Catanzaro, Italy; 4 PhD School of History and Philosophical-social Sciences, University of Rome Tor Vergata, Via Orazio Raimondo 18, 00173 Rome, Italy

**Keywords:** Nutrigenetics, FTO, Mediterranean diet, Body composition

## Abstract

**Background:**

The Mediterranean diet (MeD) plays a key role in the prevention of obesity. Among the genes involved in obesity, the Fat mass and obesity-associated gene (FTO) is one of the most known, but its interaction with MeD remained uncertain so far.

**Methods:**

We carried out a study on a sample of 188 Italian subjects, analyzing their FTO rs9939609 alleles, and the difference in body composition between the baseline and a 4-weeks nutritional intervention. The sample was divided into two groups: the control group of 49 subjects, and the MeD group of 139 subjects.

**Results:**

We found significant relations between MeD and both variation of total body fat (ΔTBFat) (p = 0.00) and gynoid body fat (p = 0.04). ∆TBFat (kg) demonstrated to have a significant relation with the interaction diet-gene (p = 0.04), whereas FTO was associated with the variation of total body water (p = 0.02).

**Conclusions:**

MeD demonstrated to be a good nutritional treatment to reduce the body fat mass, whereas data about FTO remain uncertain. Confirming or rejecting the hypothesis of FTO and its influence on body tissues during nutritional treatments is fundamental to decide whether its effect has to be taken into consideration during both development of dietetic plans and patients monitoring.

*Trial Registration* ClinicalTrials.gov Id: NCT01890070. Registered 01 July 2013, https://clinicaltrials.gov/ct2/show/NCT01890070

## Background

The Mediterranean diet (MeD) is considered one of the healthiest dietetic pattern in the world [1]. It is characterized by a high consumption of olive oil, fish, fruits, legumes, vegetables, unrefined cereals, a moderate consumption of wine and dairy products, and a low consumption of non-fish meat products [2]. According to several studies, thanks to its peculiar distribution of macro and micronutrients, this dietary model plays a key role in the prevention of a wide number of chronic diseases, such as cardiovascular diseases (CDVs), diabetes and metabolic syndrome [3–5], making an important contribution to the weight loss, especially if it is associated with energy restriction and constant physical activity [6]. In view of above, it is worth recalling that this dietary models can also prevent and treat obesity [7], a disease, usually classified through that provides alterations in body composition like abnormal or excessive fat accumulation [8–10]. This medical condition is caused by a several numbers of co-factors, such as psychological problems, sedentary life and incorrect dietetic habits [11, 12], and it is an important risk factor for all those aforementioned diseases fought by the Mediterranean diet [13]. Therefore, in this context, it is understandable why this dietary model plays a key role in the health protection.

Nevertheless, independently from the dietetic habits, also genetics play a key role in the development of obesity [14]. In the human genome, a several number of genes are involved in the obesity, and Fat mass and obesity-associated gene (FTO) is surely one of the most important. This gene, widely expressed in several fetal and adult tissues, is located on chromosome 16 (16q12.2), and encodes for the enzyme alpha-ketoglutarate-dependent dioxygenase [15]. This enzyme is implied in the regulation of both the control of adipocyte thermogenesis and differentiation, contributing considerably to the body fat accumulation [16]. Furthermore, it contributes to the regulation of energy homeostasis and metabolic rate [17], increasing also the food intake [18]. Finally, among the other activities, is implicated in the repairs of alkylated DNA and RNA via oxidative demethylation [19]. Among the several variants of this gene, FTO rs9939609 is one of the best-know. It is located in the first intron of the gene and, in 2007, it was for the first time associated with the body mass [20]. Subsequently, further studies demonstrated that the allele A of FTO rs9939609 is related to both a higher body mass index and body circumferences [21, 22], and, among different ethnicities, also Italian population demonstrated the same results [23]. Furthermore, this SNP is included among the genetic etiological factors in the development of both metabolic syndrome and Type-2 diabetes [24, 25], and allele A was also associated with a higher fat and lean mass [26, 27]. In a PREDIMED substudy, it was observed that homozygous subjects for A allele had the highest baseline body weight, but also the lowest body weight gain after 3 years of Mediterranean-style intervention, compared to TT genotype. However, no interaction between nutritional intervention and the polymorphism was found [28, 29]. On the other hand, Ortega-Azorín et al. [30] demonstrated consistent gene-diet interactions between FTO rs9939609 and Melanocortin-4 Receptor (MC4R) rs17782313 genes and to the Mediterranean diet adherence in the type 2 diabetes risk. The same effect was observed in obese phenotypes in Iranian population. Subjects with minor allele carriers of FTO variants rs9939973, rs8050136, rs1781749, and rs3751812 had a lower risk of obesity when they had a higher Mediterranean dietary score compared to wild-type homozygote genotype carriers [31].

In the light of these observations the objective of this study was to investigate about the possible influence of FTO rs9939609 variant on both weight loss and modification of body composition in Italian subjects, after having followed a 4-weeks dietetic intervention based on the Mediterranean model, in order to see if this polymorphism could influence the response to a specific dietary treatment.

## Methods

### Study design and subjects

The study was carried out between January 2017 and March 2018 at the Section of Clinical Nutrition and Nutrigenomics, Department of Biomedicine and Prevention of the University of Rome Tor Vergata. The initial sample recruited was composed of 300 subjects, who came up for the first time for a nutritional-medical checkup at the Clinical Nutrition and Nutrigenomic Section at the University of Rome Tor Vergata. To be eligible, each individual had to belong to the Caucasian race, to be Italian and older than 16 years old. Furthermore, for each subject, the medical assessment was evaluated before and after the application of prescribed nutritional treatments. We have pooled the sample in two groups, 150 subjects followed, for a period of 4 weeks, a Mediterranean diet (MeD), whereas the remaining 150 were allocated, within the same period, in the control group (CTR). The randomization and allocation of the study participants was performed using the IBM SPSS 21.0 for Windows (IBM Corp., Armonk, NY, USA). All the individuals included in the study approved their participation by learning and signing the informed consent, which was drawn up in accordance with the commissariat of the Ethics Committee of Medicine, University of Rome Tor Vergata and with the Helsinki Declaration of 1975 as revised in 1983. Trial Registration: this protocol has been registered by ClinicalTrials.gov, ID: NCT01890070.

### Anthropometric, bioimpendance analysis, and dual-energy X-ray absorptiometry

After a 12-h overnight fasting, all subjects underwent anthropometric evaluation. All the individuals were instructed to take off their clothes and shoes before undergoing the measurements. Body weight was assessed with balance scale to the nearest 0.1 kg (Invernizzi, Rome, Italy). Height was evaluated using a stadiometer (Invernizzi, Rome, Italy) to the nearest 0.1 cm. BMI was calculated using the formula ${\text{BMI}}\, = \,{\text{body weight}}/{\text{height}}^{ 2} \left( {{\text{kg}}/{\text{m}}^{ 2} } \right)$. Waist, hip, neck and abdomen circumferences were assessed using a flexible steel metric tape to the nearest 0.5 cm, according to the International Society for the Advancement of Kin anthropometry protocol and National Institute of Health Guidelines [32].

Body composition analysis was performed using both dual-energy X-ray absorptiometry (DXA) (I-DXA, GE Medical Systems, Milwaukee, WI, USA) and bioelectrical impedance analysis (BIA 101S, Akern/RJL Systems, Pontassieve, Florence, Italy). DXA was carried out to evaluate total, android and gynoid, of fat mass percentage (FM%), fat mass (FM) and lean mass (LM) in Kg. Total fat mass percentage (Total FM%) was calculated as Total body fat mass (Total FM) divided by the total mass of all tissues (Total LM), including the total body bone (TBBone), as the following: ${\text{Total FM}} \% \, = \,\left( {{\text{Total FM}}/\left( {{\text{Total FM}}\, + \,{\text{Total LM}}\, + \,{\text{TBBone}}} \right)} \right)\, \times \, 100$ [33]. Bioelectrical impedance analysis was carried out to evaluate resistance (R), reactance (Xc), phase angle (PA), hydration, exchange Na/K, total body water (TBW), extracellular water (ECW), intracellular water (ICW), body cell mass (BCM), body cell mass index (BCMI). Finally, waist/hip ratio (WHR) was analyzed and evaluated according to the clinical risk thresholds, equivalent to WHR > 0.9 for men and WHR > 0.85 for women [34].

According to De Lorenzo et al. [10], we categorized our population based on phenotype classification through BMI and TBFat% as follows: underweight (UW) (BMI < 18.50); normal-weight (NW) (18.50 ≤ BMI < 25 or BMI ≥ 25 but Total TBFat% lower than 30% for women and 25% for men); normal weight obese (NWO) (18.50 ≤ BMI < 25 and TBFat% higher than 30% for women and 25% for men); Preobese (PreOb) (25 ≤ BMI < 30 and TBFat% higher than 30% for women and 25% for men); Obesity I (30 ≤ BMI < 35); Obesity II (35 ≤ BMI < 40); Obesity III (BMI ≥ 40).

### DNA isolation and RTq-PCR analysis

The phenol–chloroform extraction described by Barker et al. [35] was used to extract the Genomic DNA, which was collected, in turn, via saliva swab. To prepare the gDNA for the genotyping, a two allele-specific fluorescent probe including a PCR primer pair (TaqMan SNP Genotyping Assays, Life Technologies, CA, USA) and a master mix including dNTPs and Taq DNA Polymerase (TaqPath ProAmp Master mix Life Technologies, CA, USA) were used. The FTO rs9939609 context sequence was the following: GGTTCCTTGCGACTGCTGTGAATTT [A/T] GTGATGCACTTGGATAGTCTCTGTT. Finally, SNP genotyping assessment was executed using a Real-Time PCR analysis (Applied Biosystems StepOnePLus Real-Time PCR, Life Technologies, CA, USA), according to the manufacturer’s instructions.

### Dietary assessments

At baseline subjects food intake was assessed with a 3-day diet records completed for 2 weekdays and 1 weekend day [36]. Participants were instructed to record weight and/or measures of foods and beverages consumed. Diet records were reviewed to clarify the amounts of foods ingested. The estimated intake of macronutrients was calculated by using Dietosystem dietary software (DS Medica S.r.l., Milan, Italy).

### Dietary intervention

In MeD intervention, an isocaloric Mediterranean diet in which the daily macronutrients intake was distributed as follows: 55% of carbohydrates, 20% of protein (> 50% of vegetable derivation), < 25% of lipids (on total daily energy intake: saturated fat < 10%, 6–10% polyunsaturated fatty acids (PUFA), n-6/n-3 PUFA ratio of 3:1, 15% of monounsaturated fatty acids (MUFA); < 1% trans-fatty acids) and 25 g of fiber.

For each subject, the energy intake was calculate according to the estimation of the rest energy expenditure (REE), which was determined using the Weir Formula:

$${\text{REE}}\, = \,\left[ {\left( { 3. 9 4\, \times \,{\text{VO}}^{ 2} } \right)\, + \,\left( { 1. 10 6 { } \times {\text{ VCO}}^{ 2} } \right)} \right] \, \times { 1}. 4 4$$

[37], in which VO^2^ and VCO^2^ were calculated as follows:

VO^2^ = Total LM DXA (kg) × 5 for females, VO^2^ = Total LM DXA (kg) × 4.5 for males and VCO^2^ = VO^2^ × 0.85 [38].

The REE was multiplied for the proper physical activity level (PAL) according to the WHO and Food and Agriculture Organization of the United Nations (FAO) recommendations [39].Conversely, in the control group, the subjects did not follow any specific diet, but they have received general recommendations on healthy nutritional habit and were only monitored, even though also for these subjects REE and the energy intake were evaluated as aforementioned.

### Analysis of blood sample

Blood samples, taken after a 12-h overnight fast, were collected in sterile tubes containing EDTA (Vacutainer^®^) and put on ice. Plasma, after being separated by centrifugation (1600 rpm, at 4 °C for 10 min), was removed, aliquoted and stored at − 80 °C. All clinical chemistry analyses, except serum lipid plasma and glucose analysis, were performed using an ADVIA^®^1800 Chemistry System (Siemens Healthcare), following standard procedures [40]. Plasma glucose concentrations were measured through the glucose oxidase method and automated glucose analyzer (COBAS INTEGRA 400, Roche Diagnostics, Indianapolis, IN, USA); serum lipid profile constituents were evaluated by standard enzymatic colorimetric techniques (Roche143 Modular P800, Roche Diagnostics, Indianapolis, IN, USA).

### Statistical analysis

The SNP-HWE program was used to calculate the Hardy-Weinberg equilibrium (HWE) for FTO rs9939609, and the result was tested using the χ^2^ analysis [41]. To analyze the sample, the subjects were divided into carrier/non-carrier (carrier for A allele vs homozygous T) and into MeD and CTR (Mediterranean diet vs Control group). The Kolmogorov–Smirnov test was used to analyze the distribution of variables, and data were normalized according to Z-score transformation. T-test analysis was performed between A carriers and TT genotype at baseline, as well as ANOVA one way analysis, adjusted with Bonferroni post hoc analysis, between the genotype/treatment groups in order to see significant differences among groups (p < 0.05). Change (Δ) in body weight (kg), BMI, neck, waist, abdomen and hip circumferences (cm), WHR, R, Xc, BCM (kg), Na/K, TBW (L), ECW (L), ICWb (L), BCMI, Android Bfat (%), Gynoid Bfat (%), TBFat (%), Android Bfat (Kg), Gynoid Bfat (Kg), TBFat (Kg), Android BLean (kg), Gynoid BLean (kg), TBLean (kg) and REE were calculated by subtracting measurements recorded before the beginning of the nutritional treatments, from the measurement recorded after the completion of the nutritional intervention. In order to compare the differences in mean of the aforementioned values, respectively for A carriers and TT genotype, gene, diet and gene–diet interaction analyses were carried out using a Generalized Linear Model (linear GLM), adjusted for sex and age. TBFat (kg) was selected as a parameter to calculate minimum sample size. The minimum sample size was calculated using a two-tailed one-sample Student’s t-test, considering:(i) TBFat to be detected between baseline and MED|δ| = − 3.86 kg; (ii) SD of the paired differences SD = 6.38 kg, (iii) type I error probability α = 0.05 and power 1 − β = 0.95. The result was a minimum sample size of 36 for MED and 12 for CTR group. Significance was set as p < 0.05 and the statistical analysis was performed using IBM SPSS 21.0 for Windows (IBM Corp., Armonk, NY, USA).

## Results

### Population characteristics

**Fig. 1:**
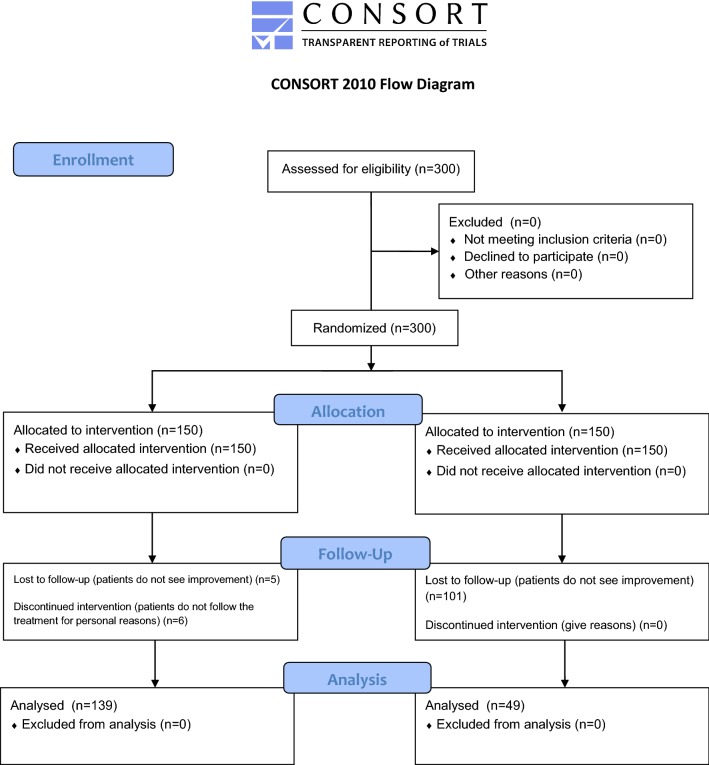
Study design

The enrolled 300 subjects met the inclusion criteria and nobody declined to participate. Subjects were equally randomized allocated in MeD group and CTR group. During this clinical trial, 11 subjects from MeD group and 101 subjects from CTR group abandoned the study as specified in Fig. 1. In fact, 5 subjects form the MeD group abandoned the study for poor weight loss results and 6 subjects for poor adherence to the diet therapy. On the other hand, in the CTR group 101 subjects abandoned the study since they did not see any kind of health benefits or improvement. The final sample analyzed consisted of 188 patients, divided as follow: 139 subjects in MeD group and 49 for CTR group. These patients successfully participated and completed the study protocol.

In our sample, the HWE was respected (p > 0.05). The comprehensive description of the whole sample population at baseline can be seen in Table 1. All results were stated as mean and standard deviation. The average age of the individuals was 46.83 ± 15.01 years, 62.2% females and 37.8% males (Table 1). According to the statistical analysis, at baseline, carriers and no carriers differs significantly only for age, diastolic blood pressure, R, basophils count and unsaturated fatty acids and polyunsaturated fatty acids intake (p < 0.05) (Table 1). Genotype frequencies of our individuals (TT: 0.310; AA: 0.190; AT: 0.500) are similar to the ones shown in TSI population (TT: 0.327, AA: 0.252, AT: 0.421), and the same result was demonstrated about the allele frequencies, similar between TSI (T: 0.537; A: 0.463) and our sample (T: 0.550; A: 0.450) (Table 2). The average BMI of the subject was 29.39 ± 6.99, and the average Total FM (kg) was 37.3 ± 9.76. Finally, in order to have a detailed description of the sample, in Table 3 the individuals, at the baseline, were divided by genotype and categorized according to phenotype classification.

**Table 1:** Descriptive characteristics of study population

	Total	TT genotype	A carriers
Age (years)	46.83 (± 15.01)	43.29 (± 16.04)^a^	48.36 (± 14.34)^a^
Systolic BP (mm Hg)	126.14 (± 17.03)	127.96 (± 20.80)	125.42 (± 15.41)
Distolic BP (mm Hg)	86.62 (± 19.45)	94.42 (± 23.64)^a^	83.30 (± 16.48)^a^
Height (cm)	165.85 (± 9.04)	166.17 (± 9.52)	165.71 (± 8.86)
Weight (kg)	80.9 (± 21.14)	77.83 (± 20.47)	82.23 (± 21.36)
BMI (kg/m^2^)	29.39 (± 6.99)	28.09 (± 6.68)	29.95 (± 7.08)
Neck circumference (cm)	39.47 (± 4.34)	38.71 (± 3.80)	39.70 (± 4.48)
Waist circumference (cm)	90.95 (± 16.38)	87.63 (± 16.45)	92.23 (± 16.25)
Abdomen circumference (cm)	103.31 (± 15.75)	98.71 (± 14.81)	104.70 (± 15.85)
Hip circumference (cm)	106.49 (± 12.09)	103.38 (± 9.84)	107.68 (± 12.68)
WHR	0.85 (± 0.11)	0.84 (± 0.11)	0.86 (± 0.11)
R	505.78 (± 96.34)	538.67 (± 83.75)^a^	492.62 (± 98.31)^a^
Xc	55.56 (± 13.15)	57.14 (± 10.34)	54.93 (± 14.13)
PA	6.42 (± 1.33)	6.24 (± 1.11)	6.49 (± 1.41)
BCM (kg)	30.27 (± 8.37)	28.03 (± 7.16)^a^	31.18 (± 8.68)^a^
Na/K	0.94 (± 0.16)	0.97 (± 0.12)	0.93 (± 0.17)
BCM (%)	55.75 (± 6.97)	54.46 (± 4.92)	56.30 (± 7.66)
TBW (L)	40.36 (± 9.52)	38.44 (± 8.60)	41.14 (± 9.81)
ECW (L)	17.76 (± 4.46)	17.23 (± 3.94)	17.97 (± 4.66)
ICW (L)	22.76 (± 6.45)	21.43 (± 5.62)	23.33 (± 6.73)
BCMI	12.5 (± 6.58)	11.68 (± 5.64)	12.83 (± 6.92)
Android FM%	43.19 (± 12.54)	42.18 (± 11.70)	43.55 (± 12.87)
Gynoid FM%	41.03 (± 10.93)	40.90 (± 10.32)	41.09 (± 11.21)
Total FM%	37.3 (± 9.76)	36.56 (± 8.83)	37.62 (± 10.15)
Android FM (kg)	3.13 (± 2.02)	2.84 (± 1.79)	3.25 (± 2.10)
Gynoid FM (kg)	5.45 (± 2.3)	5.15 (± 2.03)	5.57 (± 2.39)
Total FM (kg)	29.93 (± 14.88)	28.48 (± 13.80)	30.56 (± 15.33)
Android LM (kg)	3.25 (± 0.88)	3.31 (± 0.95)	3.23 (± 0.85)
Gynoid LM (kg)	6.88 (± 1.82)	6.92 (± 1.95)	6.86 (± 1.77)
Total LM (kg)	47.6 (± 11.03)	47.31 (± 11.69)	47.73 (± 10.80)
VO^2^	231.81 (± 46.56)	228.71 (± 46.22)	234.23 (± 46.14)
VCO^2^	197.04 (± 39.57)	194.40 (± 39.29)	199.10 (± 39.22)
REE	1628.52 (± 326.88)	1602.65 (± 322.62)	1647.16 (± 324.44)
Glycemia (mg/dL)	92.77 (± 15.81)	93.83 (± 15.11)	92.34 (± 16.19)
Insulin (μ/μmL)	8.23 (± 4.4)	6.80 (± 4.50)	9.08 (± 4.25)
Tot cholesterol (mg/dL)	197.87 (± 43.13)	185.36 (± 47.98)	202.34 (± 40.70)
HDL (mg/dL)	54.5 (± 15.86)	58.46 (± 16.82)	53.10 (± 15.40)
TG (mg/dL)	109.31 (± 50.71)	95.40 (± 49.41)	114.22 (± 50.61)
LDL (mg/dL)	122.21 (± 30.01)	111.17 (± 33.38)	126.05 (± 28.00)
GOT/AST (uL)	22.16 (± 9.05)	21.91 (± 12.63)	22.24 (± 7.64)
GPT/ALT (uL)	24.67 (± 12.5)	23.81 (± 12.92)	24.95 (± 12.44)
Basophils (1000/μL)	0.02 (± 0.03)	0.03 (± 0.03)^a^	0.02 (± 0.02)^a^
Basophils (%)	0.53 (± 0.36)	0.67 (± 0.48)	0.44 (± 0.20)
Eosinophils (1000/μL)	0.23 (± 0.37)	0.36 (± 0.64)	0.17 (± 0.10)
Eosinophils (%)	3.07 (± 2.01)	3.81 (± 2.87)	2.74 (± 1.40)
HCT (%)	41.87 (± 3.2)	41.83 (± 2.70)	41.89 (± 3.43)
Hemoglobin (g/dL)	14.11 (± 1.32)	14.05 (± 1.11)	14.14 (± 1.41)
Lymphocytes (1000/μL)	2.19 (± 1.06)	2.08 (± 0.57)	2.24 (± 1.22)
Lymphocytes (%)	32.75 (± 7.3)	33.08 (± 7.69)	32.62 (± 7.22)
MCH (pg)	29.71 (± 2.1)	29.73 (± 1.52)	29.70 (± 2.32)
MCHC (g/dL)	33.66 (± 1.43)	33.74 (± 1.42)	33.64 (± 1.45)
MCV (fL)	88.3 (± 4.42)	88.98 (± 4.63)	87.82 (± 4.33)
Monocytes (1000/μL)	0.47 (± 0.18)	0.46 (± 0.15)	0.47 (± 0.19)
Monocytes (%)	7.29 (± 2.03)	7.21 (± 1.46)	7.32 (± 2.24)
Neutrophiles (1000/μL)	3.64 (± 1.15)	3.59 (± 1.04)	3.67 (± 1.25)
Neutrophiles (%)	56.12 (± 7.99)	55.69 (± 8.52)	56.44 (± 7.78)
Plateles (1000/μL)	205.83 (± 36.84)	210.17 (± 39.32)	202.94 (± 35.96)
RBC (million/μL)	4.74 (± 0.43)	4.68 (± 0.41)	4.78 (± 0.44)
RDW-CV	12.79 (± 3.74)	10.92 (± 3.45)	13.62 (± 3.60)
WBC (1000/μL)	7.21 (± 4)	7.79 (± 5.55)	6.86 (± 2.81)
ESR (mm)	31.03 (± 68.58)	36.00 (± 86.37)	29.14 (± 62.24)
Fibrinogen (mg/dL)	290.29 (± 168.28)	351.40 (± 85.57)	264.10 (± 193.31)
Uric acid (mg/dL)	8.84 (± 9.14)	10.64 (± 11.23)	8.14 (± 8.26)
CRP (mg/dL)	1.79 (± 3.13)	1.32 (± 2.04)	1.95 (± 3.45)
Kcal	1721.86 (± 731.69)	1730.05 (± 823.19)	1711.23 (± 636.34)
Proteins (g)	83.18 (± 27.04)	82.16 (± 35.01)	84.51 (± 12.22)
Proteins (%)	19.33 (± 3.95)	19.86 (± 4.60)	18.64 (± 3.00)
Carbohydrates (g)	223.05 (± 106.41)	209.86 (± 131.30)	240.19 (± 64.14)
Carbohydrates (%)	46.88 (± 7.63)	46.19 (± 8.43)	47.78 (± 6.78)
Simple carbohydrates (g)	75.83 (± 33.01)	75.06 (± 43.41)	76.82 (± 12.23)
Complex carbohydrates (g)	128.72 (± 55.39)	125.68 (± 64.34)	132.68 (± 44.16)
Lipids (g)	62.17 (± 26.78)	58.91 (± 30.67)	66.40 (± 21.54)
Lipids (%)	30.84 (± 6.64)	30.50 (± 5.76)	31.29 (± 7.95)
Total cholesterol (mg)	218.90 (± 90.23)	218.67 (± 106.36)	219.20 (± 69.40)
Saturated fatty acids (g)	17.02 (± 10.07)	15.84 (± 11.65)	18.55 (± 7.89)
Unsaturated fatty acids (g)	8.80 (± 4.07)	7.21 (± 3.72)^a^	10.87 (± 3.69)^a^
Monounsaturated fatty acids (g)	52.13 (± 132.35)	72.95 (± 176.00)	25.06 (± 8.58)
Polyunsaturated fatty acids (g)	9.11 (± 4.25)	7.21 (± 4.04)^a^	11.42 (± 3.39)^a^
Fiber (g)	22.63 (± 9.92)	20.50 (± 5.09)	25.39 (± 13.82)

Descriptive table of the overall study population. Data were reported as mean and standard deviation. Statistical significance (a) among A carriers and TT genotype groups at baseline were given to results with p < 0.05 through t-test analysis

*BP* blood pressure, *BMI* body mass index, *WHR* waist hip ratio, *Xc* reactance, *R* resistance, *PA* phase angle, *BCM* body cell mass, *HYDR* hydration, *Na/K* sodium–potassium exchange, *TBW* total body water, *ECW* extracellular water, *ICW* intracellular water, *BCMI* body cell mass index, *BFat* body fat, *TBFat*: total body fat, *BLean* body lean, *TBLean* total body lean, *REE* resting energy expenditure, *HDL* high-density lipoprotein, *TG* triglycerides, *LDL* low-density lipoprotein, *GOT* glutamic oxaloacetic transaminase, *AST* aspartate aminotransferase, *GPT* glutamate pyruvate transaminase, *ALT* alanine transaminase, *HCT* hematocrit, *MCH* mean corpuscular hemoglobin, *MCHC* mean corpuscular hemoglobin concentration, *MCV* mean corpuscular volume, *RBC* red blood cells, *RDW-CV* red blood cell distribution width, *WBC* white blood cells, *ESR* erythrocyte sedimentation rate, *CRP* C-reactive protein

**Table 2:** Study population allele and genotype frequencies for FTO rs9939609 compared to Tuscan Italians from Southern Europe (TSI)

FTO rs9939609			
Allele frequency	A	T	
TSI	0.46	0.54	
Study population	0.45	0.55	
Genotype frequency	AA	AT	TT
TSI	0.20	0.46	0.34
Study population	0.19	0.50	0.31

Description of study population and genotype frequencies

**Table 3:** BMI distribution of study population according to FTO rs9939609 variant

BMI	TT	A carrier
	n = 37 (19.70%)	n = 151 (80.30%)
UW	1 (2.71%)	3 (1.99%)
NW	5 (13.51%)	21 (13.90%)
NWO	3 (8.11%)	27 (17.88%)
PreOb	10 (27.03%)	49 (32.46%)
OB I	12 (32.43%)	23 (15.23%)
OB II	5 (13.51%)	10 (6.62%)
OB III	1 (2.70%)	18 (11.92%)

Frequencies of carrier and non-carrier subjects according to the BMI. UW (BMI < 18.50); NW (18.50 ≤ BMI < 25 or BMI ≥ 25 plus TBFat% < 30% females and < 25% M); NWO (18.50 ≤ BMI < 25 plus Total TBFat% ≥ 30% females and ≥ 25% males); PreOb (25 ≤ BMI < 30 plus TBFat% ≥ 30% females and ≥ 25% males); Ob I (30 ≤ BMI < 35); Ob II (35 ≤ BMI < 40); Ob III (BMI ≥ 40). *BMI* body mass index, *UW* underweight, *NW* normal-weight, *NWO* normal-weight obese, *PreOb* pre obese, *Ob* Obese

At baseline, differences were highlighted between TT genotype and A carriers for age, DBP, BCM (kg), basophils (1000/μL), unsaturated fatty acids (g) and polyunsaturated fatty acids (g) intake (p < 0.05).

Among the genotype/treatment groups significances were found for SBP between control group TT genotype and Med group TT genotype, DBP between control group TT genotype and the other groups (control group A carriers, Med group TT genotype and Med group A carriers) and neck circumference between control group TT genotype and Med group A carriers, control group A carriers and Med group TT genotype and Med group A carriers (p < 0.05). No other statistical significance were found at baseline between groups.

### Influences of FTO rs9939609 and nutritional intervention on BMI, body composition and metabolism

In this study, the GLM analysis was used to demonstrate the statistical significance between FTO rs9939609 carriers A and TT genotype together with the nutritional intervention.

According to the results, TBFat (kg) decreases dependently both of the nutritional intervention (p_D_ = 0.00) and the interaction gene-diet (p_GD_ = 0.04), showing a significant difference between MeD and CTR, but also suggesting a potential role, even though not statistically significant, of FTO rs9939609 (p_G_ = 0.06), since A carrier CTR gained weight sensibly more than TT genotype CTR, and A carrier MeD lost more weight than TT genotype MeD (MeD/TT genotype = − 3.59 ± 4.78; MeD/A carriers = − 3.97 ± 6.97; CTR/TT genotype = 1.93 ± 5.54; CTR/A carriers = 6.11 ± 9.60) (Table 4). Furthermore, MeD group, regardless of FTO rs9939609 (p_G_ = 0.70; p_GD_ = 0.32) (Table 4), lost a higher amount of Gynoid BFat (%) compared to CTR (p = 0.04) (MeD/TT genotype = − 2.94 ± 5.14; MeD/A carriers = − 1.67 ± 3.51; CTR/TT genotype = 0.03 ± 1.26; CTR/A carriers = − 0.42 ± 1.74) (Table 4).

**Table 4:** Anthropometric, body composition and REE analysis for FTO rs9939609 A carriers vs TT genotype in MeD and CTR groups

	TT genotype		A carriers		P main effect of FTO rs9939609	P main effect of diet	P main effect gene-diet interaction
	MeD	CTR	MeD	CTR			
	(n = 40)	(n = 17)	(n = 99)	(n = 32)			
Weight (kg)					0.85	0.43	0.87
Baseline	79.63 (21.56)	73.33 (17.26)	83.29 (22.73)	78.73 (15.87)			
Change	− 3.41 (6.47)	− 1.27 (3.89)	− 2.25 (11.79)	− 0.62 (1.26)			
BMI					0.92	0.33	0.81
Baseline	29.03 (7.39)	25.74 (3.64)	30.4 (7.35)	28.50 (6.03)			
Change	− 1.28 (2.34)	− 0.30 (0.82)	− 0.85 (4.27)	− 0.18 (0.80)			
Neck circumference (cm)					0.50	0.78	0.37
Baseline	40.54 (3.39)	36.75 (3.28)	41.03 (4.19)	36.88 (3.76)			
Change	− 0.60 (0.42)	− 0.40 (0.96)	− 1.09 (2.13)	− 0.07 (1.12)			
Waist circumference (cm)					0.80	0.87	0.86
Baseline	88.17 (19.18)	86.81 (11.66)	94.14 (17.25)	87.7 (12.72)			
Change	− 1.10 (3.03)	− 0.78 (1.82)	− 1.91 (7.55)	− 1.10 (2.34)			
Abdomen circumference (cm)					0.87	0.97	0.28
Baseline	104.92 (18)	93.74 (9.66)	107.49 (15.9)	99.48 (14.6)			
Change	− 0.80 (1.48)	− 1.04 (3.46)	− 3.73 (6.51)	− 1.13 (2.89)			
Hip circumference (cm)					0.93	0.70	0.76
Baseline	103.8 (11.16)	102.72 (7.68)	107.68 (13.01)	107.67 (12.13)			
Change	− 1.90 (4.04)	− 1.38 (3.55)	− 2.51 (3.93)	− 1.44 (3.29)			
WHR					0.80	0.89	0.89
Baseline	0.84 (0.12)	0.84 (0.08)	0.87 (0.12)	0.82 (0.08)			
Change	0.00 (0.02)	0.00 (0.03)	0.00 (0.07)	0.00 (0.03)			
R					0.10	0.98	0.87
Baseline	533.35 (88.18)	545.31 (80.2)	484.66 (107.09)	507.77 (78.38)			
Change	− 3.00 (22.26)	0.24 (33.1)	20.11 (59.77)	25.91 (42.27)			
Xc					0.38	0.32	0.38
Baseline	55.8 (11.45)	58.81 (8.83)	54.98 (15.84)	54.84 (10.33)			
Change	0.00 (4.76)	− 4.53 (6.69)	− 2.2 (10.33)	− 2.13 (8.13)			
PA					0.73	0.45	0.58
Baseline	6.23 (1.06)	6.24 (1.22)	6.62 (1.54)	6.22 (1.07)			
Change	0.10 (0.34)	− 0.40 (0.79)	− 0.47 (1.57)	− 0.53 (0.94)			
BCM (kg)					0.17	0.91	0.94
Baseline	27.29 (6.39)	28.95 (8.13)	31.99 (9.68)	29.66 (6.28)			
Change	− 0.33 (0.98)	− 0.54 (3.51)	− 2.62 (7.63)	− 2.75 (4.26)			
Na/K					0.64	0.66	0.53
Baseline	0.97 (0.12)	0.98 (0.13)	0.91 (0.18)	0.96 (0.13)			
Change	0.03 (0.1)	0.06 (0.1)	0.06 (0.17)	− 0.12 (0.63)			
TBW (L)					0.02*	0.48	0.47
Baseline	38.36 (8.55)	38.54 (8.94)	41.76 (10.99)	39.81 (6.57)			
Change	− 0.83 (1.63)	0.70 (3.04)	− 1.88 (4.35)	− 1.75 (3.28)			
ECW (L)					0.81	0.43	0.56
Baseline	17.23 (4.06)	17.22 (3.9)	18.01 (5.27)	17.89 (3.04)			
Change	− 0.25 (1.5)	2.00 (3.26)	2.16 (6.74)	2.42 (4.07)			
ICW (L)					0.65	0.92	0.81
Baseline	21.12 (5.16)	17.22 (3.9)	23.75 (6.97)	20.59 (4.05)			
Change	− 0.25 (0.74)	− 0.54 (1.8)	− 1.89 (4.85)	− 0.95 (1.79)			
BCMI					0.70	0.91	0.52
Baseline	12.77 (7.02)	10.11 (1.96)	13.9 (8.08)	10.56 (1.94)			
Change	− 0.10 (0.34)	− 0.34 (1)	− 2.40 (6.81)	− 0.85 (1.23)			
Android Bfat (%)					0.60	0.09	0.93
Baseline	44.92 (12.45)	37.34 (8.78)	44.88 (12.77)	39.79 (12.61)			
Change	− 4.52 (6.01)	− 1.00 (2.16)	− 2.96 (4.64)	0.15 (1.7)			
Gynoid Bfat (%)					0.70	0.04*	0.32
Baseline	43.51 (10.63)	36 (7.85)	41.8 ( 77(11.76)	39.07 (9.37)			
Change	− 2.94 (5.14)	0.03 (1.26)	− 1.67 (3.51)	− 0.42 (1.74)			
TBFat (%)					0.79	0.08	0.94
Baseline	37.92 (9.45)	33.16 (6.05)	38.08 (10.34)	36.14 (9.52)			
Change	− 2.74 (3.45)	− 0.19 (1.14)	− 2.01 (3.65)	0.31 (0.9)			
Android Bfat (kg)					0.89	0.27	0.91
Baseline	3.02 (1.96)	2.32 (1.08)	3.3 (2.17)	2.78 (1.39)			
Change	− 0.47 (0.53)	− 0.04 (0.27)	− 0.45 (1.18)	− 0.12 (0.63)			
Gynoid Bfat (kg)					0.49	0.84	0.40
Baseline	5.57 (2.19)	4.37 (1.45)	5.49 (2.58)	5.78 (1.79)			
Change	− 0.62 (0.85)	− 0.46 (1.25)	− 0.36 (2.82)	− 0.94 (2.25)			
TBFat (Kg)					0.06	0.00*	0.04*
Baseline	31.34 (14.5)	21.32 (8.66)	32.85 (15.56)	23.24 (12.11)			
Change	− 3.59 (4.78)	1.93 (5.54)	− 3.97 (6.97)	6.11 (9.60)			
Android BLean (kg)					0.97	0.32	0.12
Baseline	3.32 (1.01)	3.31 (0.86)	3.2 (0.91)	3.31 (0.62)			
Change	− 0.22 (0.87)	− 0.08 (0.15)	0.11 (0.6)	− 0.08 (0.27)			
Gynoid BLean (kg)					0.49	0.92	0.91
Baseline	6.77(1.86)	7.24(2.16)	6.79(1.9)	7.08(1.26)			
Change	− 0.20(1.54)	− 0.18(0.31)	− 0.56(5.66)	0.01(0.33)			
TBLean (kg)					0.92	0.63	0.87
Baseline	47.67(11.77)	46.47(11.84)	47.86(11.37)	47.28(8.87)			
Change	− 1.23(6.82)	− 0.53(1.05)	− 0.56(5.66)	− 0.61(2.08)			
REE					0.77	0.27	0.49
Baseline	1639.92 (409.68)	1664.96 (217.89)	1628.13 (347.37)	1583.46 (313.51)			
Change	− 48.13 (327.01)	− 31.44 (140.89)	− 44.01 (220.32)	− 7.49 (34.75)			

Relationship between FTO rs9939609 A carriers and TT genotype in body composition and metabolism, according to MeD and CTR groups. Statistical significance (*) were given to results with p < 0.05 through GLM analysis. *BMI* body mass index, *WHR* waist hip ratio, *R* resistance, *Xc* reactance, *PA* phase angle, *HYDR* hydration, *NA/K* sodium–potassium exchange, *TBW* total body water, *ECW* extracellular water, *ICW* intracellular water, *BFat* body fat, *TBFat* total body fat, *BLean* body lean, *TBLean* total body lean, *REE* resting energy expenditure

The bioelectrical impedance analysis highlighted that TBW, was significantly reduced in A carriers compared to TT genotype (p_G_ = 0.02), independently of nutritional intervention (p_D_ = 0.48; p_GD_ = 0.47) (MeD/TT genotype = − 0.83 ± 1.63; MeD/A carriers = − 1.88 ± 4.35; CTR/TT genotype = 0.70 ± 3.04; CTR/A carriers = − 1.75 ± 3.28) (Table 4). All the other results were not statistically significant (p > 0.05).

## Discussion

Among the different dietetic patterns, the Mediterranean diet is surely one of the healthiest in the world [1]. In fact, several studies demonstrated its capability to prevent different cardiovascular and metabolic diseases, such as metabolic syndrome and type-2 diabetes [2, 4, 5]. Moreover, this type of dietetic model turns out to be fundamental both in preventing and treating the obesity [6, 42], which is becoming one of the most widespread medical condition in the world, since recent statistics demonstrated that, by 2030, the 20% of the global adult population will be obese, and the 38% will be overweight [43]. Nevertheless, also genetics plays an important role in the development of the obesity [14]. So far, a wide number of genes are associated with the body composition, and one of the most studies is certainly FTO. Several variants of this gene were associated with the BMI, and FTO rs9939609 is certainly one of the most known [44, 45]. In fact, its allele A is highly related with higher BMI [20, 46], fat and lean mass [26, 27]. On the contrary, for the time being, several studies demonstrated that, during a dietetic treatment, FTO rs9939609 do not seem to influence the weight loss [28, 47]. In view of above, we have conducted this study in order to analyze whether and how FTO rs9939609 variant influence both weight loss and body composition in Italian patients, within a Mediterranean dietetic treatment.

According to our results, TBW is highly influenced by FTO. In fact, analyzing the alteration of this value in each group (Table 4), it is possible to see that A carriers lost a higher quantity of body water compared to TT genotype (MeD/TT genotype = − 0.83 ± 1.63; CTR/TT genotype = 0.70 ± 3.04; MeD/A carriers = − 1.88 ± 4.35; CTR/A carriers = − 1.75 ± 3.28) (Table 4), whilst no difference due to MeD intervention was found. This result may be a confounding factor in the weight loss, since it influences the decreasing of both BMI and body weight during nutritional treatments, overestimating the effective fat mass loss. Analyzing the results of body composition evaluated via DXA, both dietetic treatment and interaction diet-gene influence the total body fat mass, whilst FTO alone did not demonstrate the same effect. Moreover, Gynoid BFat% declining was highly related to the dietetic therapy, confirming that MeD treatment had an effect on body composition, confirming its capabilities, already demonstrated in several studies [48, 49], to reduce body fat, giving its important contribution against all the diseases related to the body fat mass. In our sample, the difference between the two nutritional treatments is highly considerable, since both MeD groups lost weight in a similar manner. It is also important to highlight that, in our sample, FTO did not influence the outcome of the nutritional treatment, thus confirming the results of previous studies [28, 50]. These results might mean that, during a dietetic therapy, this SNP should not be taken into consideration, since it does not influence the outcome of both body composition and anthropometric measurements. However, the fact that also the interaction gene-diet had an effect on the TBFat suggests that, even though slightly, FTO might influence the outcome of the Mediterranean diet on this specific value, as previously proposed by another study [51]. Nonetheless, analyzing our results, it can be assumed that this statistical significance may be given by the incredible amount of TBFat gained by the A carriers/CTR group, thus suggesting an interesting role of this SNP in the development and preservation of body fat mass outside periods of nutritional therapies, as previously assumed by studies on murine models [18, 52]. Considering these results, we suggest that understanding whether and how FTO, along with other genes or alone, influence the fat mass loss during nutritional treatments could help us to draft better clinical pictures of patients, and prevent difficulties of weight loss due to genetic factors. Moreover, both nutritional treatment and FTO alleles did not influence the lean mass, which, in contrast to the fat mass, remained almost steady within the studied period in all the analyzed groups. Finally, according to the statistical significance, in our sample, FTO rs9939609 did not influence body circumferences, weight, and consequently BMI. Nevertheless, even though in a non-significant manner, analyzing the data in Table 4, it can be seen how A carriers subjects lost less weight compared to TT genotype, following a trend already noted [53], and leaving some doubt about the effective activities of this SNP during nutritional treatments.

## Conclusions

To conclude, Mediterranean diet confirmed to be useful in the prevention and treatment against obesity, since it demonstrated, in our sample, to reduce the body fat mass. On the contrary, data about FTO remain uncertain, and considering the importance of this SNP in the relation with BMI and body composition, further studies are needed to clarify definitely whether this SNP influences the outcome of the dietetic therapies. If FTO rs9939609 demonstrated to influence the decreasing of body tissues during nutritional treatments, its effect should be taken into consideration during both development of dietetic plans and monitoring of patients.

## Abbreviations

ALT: alanine transaminase
AST: aspartate aminotransferase
BIA: bioelectrical impedence analysis
BCM: body cell mass
BCMI: body cell mass index
BFat: body fat mass
BLean: body lean mass
BMI: body mass index
BMC: bone mineral content
BMD: bone mineral density
BP: blood pressure
CDVs: cardiovascular diseases
CRP: C-reactive protein
CTR: control group
DXA: dual-energy X-ray absorptiometry
ESR: erythrocyte sedimentation rate
ECW: extracellular water
FTO: fat mass and obesity-associated gene
GLM: generalized linear models
GPT: glutamate pyruvate transaminase
GOT: glutamic oxaloacetic transaminase
HWE: Hardy–Weinberg equilibrium
HCT: hematocrit
HDL: high-density lipoprotein
ICW: Intracellular Water
LDL: low-density lipoprotein
MCH: mean corpuscular hemoglobin
MCHC: mean corpuscular hemoglobin concentration
MCV: mean corpuscular volume
MeD: Mediterranean diet
NW: normal-weight
NWO: normal-weight obese
Ob: obese
OR: odds ratios
pD: P main effect of diet
pG: P main effect of FTO rs9939609
pGD: P main effect gene-diet interaction
PA: phase angle
PreOb: pre obese
Xc: reactance
RBC: red blood cells
RDW-CV: red blood cell distribution width
R: resistance
REE: resting expenditure rate
SNP: single nucleotide polymorphism
NA/K: sodium–potassium exchange
TSI: Toscans in Italy
TBBone: total body bone
TBFat: total body fat mass
TBLean: total body lean mass
TBW: total body water
TG: triglycerides
UW: underweight
WHR: waist hip ratio
WBC: white blood cells
WHO: World Health Organization
